# Comparative Evaluation of the Effect of Different Types of Bleaching Dentifrices on Shear Bond Strength of Orthodontic Metal Brackets Bonded by Conventional Composite to Human Teeth: An In Vitro Study

**DOI:** 10.7759/cureus.62646

**Published:** 2024-06-18

**Authors:** Ruchi Mhatre, Nitin Gulve, Shraddha Bhangare, Gajanan Garode, Amit Nehete, Shivpriya Aher Borse

**Affiliations:** 1 Department of Orthodontics and Dentofacial Orthopaedics, Mahatma Gandhi Vidyamandir's Karmaveer Bhausaheb Hiray Dental College and Hospital, Nashik, IND

**Keywords:** metal brackets, orthodontics, shear bond strength, dentifrices, bleaching

## Abstract

Aim: This study aims to evaluate and compare the effect of different types of bleaching dentifrices on the shear bond strength of orthodontic metal brackets bonded by light-cured composite adhesive to human teeth.

Materials and methods: Forty-five human premolar teeth were randomly divided into three groups, receiving the following treatments: Group 1 (control group; teeth in this group were not bleached), Group 2 (teeth in this group were treated with active oxygen bleaching dentifrice), and Group 3 (teeth in this group were treated with peroxide bleaching dentifrice). Orthodontic brackets were bonded using a light-cured composite adhesive. A universal measuring device was used to assess the shear bond strength with a crosshead speed of 0.5 mm/min. One-way ANOVA, post hoc Tukey tests, and an independent t-test were used to analyse the data.

Results: There was a highly significant difference (p≤0.001) in the mean shear bond strength of orthodontic brackets bonded to untreated teeth as compared to teeth treated with bleaching dentifrice. There was no significant difference in the mean shear bond strength of orthodontic brackets bonded to teeth treated with peroxide bleaching dentifrice or active oxygen bleaching dentifrice.

Conclusion: There was a significant reduction in the mean shear bond strength of orthodontic metal brackets when bonded to human teeth treated with bleaching dentifrices.

## Introduction

In this new age of social media, the aesthetic demands of patients have increased, leading to a surge in seeking procedures to provide a better smile. Orthodontists occasionally see patients who complain about problems with the alignment and colour of their teeth, since tooth discolouration is regarded as a significant aesthetic concern. Many orthodontic patients present with a history of dental bleaching prior to orthodontic treatment [1] to achieve the desired dental colour.

The aesthetics of a smile are adversely affected by factors like smoking, consuming foods and/or beverages that contain pigments, using products like chlorhexidine, and not practicing good oral hygiene [2]. Tooth whitening and orthodontic therapy are routine procedures to create beautiful smiles because well-aligned, white teeth give a youthful, healthy appearance [2].

Bleaching is an oxidising chemical-based procedure that affects the transmission and reflection of light, enhancing perceived whiteness. The process of bleaching teeth to make them whiter might involve mechanical, chemical, or optical methods that use abrasives and materials like whitening dentifrices to remove surface stains [3].

With the introduction of peroxide-based bleaching treatments that may be administered by patients and growing social media coverage, tooth whitening has become more and more popular. Currently, many whitening products with the promise of rapid and convenient colour alteration are available on the market, including whitening toothpaste. Due to their affordability, convenience of use, unrestricted sales, and widespread availability, whitening toothpastes have gained popularity. Peroxides are considered more effective, more conservative, less expensive, and safer when used under professional supervision [4]. Recently, toothpastes incorporating peroxide have become available. Newer ingredients are being used to improve the bleaching effect of dentifrices. One such latest ingredient is active oxygen. It shows faster action and visible results in less time.

Adults seeking orthodontic treatment are becoming more common, and more of them are likely to have had bleaching done in the past. Tooth bleaching prior to orthodontic treatment produces a considerable reduction in bond strength between the orthodontic brackets and the tooth enamel [5,6]. With the recent advances in whitening procedures, there are several options available to the patient, such as whitening strips and bleaching dentifrices. As compared to in-office bleaching, the effectiveness of these methods is dependent on the patient’s usage. Previous studies show the effect on shear bond strength of orthodontic brackets of in-office bleaching, at-home bleaching, whitening strips, and different types of whitening and bleaching dentifrices [7,8]. However, there have been no previous studies that compare the shear bond strength of orthodontic brackets after treatment with different types of bleaching dentifrices.

Therefore, the study aimed to evaluate and compare the shear bond strength of orthodontic brackets bonded to human teeth treated with peroxide bleaching dentifrice and active oxygen bleaching dentifrice. The null hypothesis tested was that there was no difference in the shear bond strength of orthodontic brackets bonded to human teeth when treated with peroxide bleaching dentifrice and active oxygen bleaching dentifrice.

## Materials and methods

This in vitro study was carried out in the Department of Orthodontics and Dentofacial Orthopaedics, Mahatma Gandhi Vidyamandir's Karmaveer Bhausaheb Hiray Dental College and Hospital, Nashik, India. The Institutional Ethical Committee of Mahatma Gandhi Vidyamandir's Karmaveer Bhausaheb Hiray Dental College and Hospital gave its approval for this research (approval number: MGV/KBHDC/988/2023-24). The sample size was calculated according to the following formula:

$$n=2 (S^2 〖(Z1+Z2)〗^2)/〖(M1-M2)〗^2$$

The sample comprises 45 extracted premolars obtained from the Department of Oral and Maxillofacial Surgery at the college. Intact teeth and teeth with sufficient root length were selected, and teeth with caries, fracture, restoration, attrition, cracks, hypoplasia, or hypocalcification were excluded.

Sample preparation

The specimens were mounted in cold-cured acrylic resin. Using a low-speed rubber cup, pumice and water were used for 10 seconds to clean the premolar buccal surface. They were then rinsed with water for 30 seconds and allowed to air dry for 15 seconds. The samples were numbered and randomly divided into three groups equally, each containing 15 samples: Group 1 (control group; teeth in this group were not bleached), Group 2 (active oxygen bleaching dentifrice; teeth in this group were treated with active oxygen bleaching dentifrice; Colgate Visible White O2 oxygenated toothpaste), and Group 3 (peroxide bleaching dentifrice; teeth in this group were treated with peroxide bleaching dentifrice; Oralvit baking soda and peroxide toothpaste).

Bleaching

For Groups 2 and 3, a pea size of the dentifrices was applied over the buccal surface, and daily tooth brushing was stimulated by brushing them in a circular motion for six minutes. The brushing treatment was performed for three days and seven days for active oxygen dentifrice and peroxide dentifrice, respectively, as per the manufacturer instructions.

Bonding procedures

The enamel surface was etched with 37% phosphoric acid gel for 30 seconds, followed by a 20-second water spray rinse and a 20-second oil-free compressed air drying. The primer (Transbond XT Primer; 3M Unitek, St. Paul, Minnesota, USA) was applied to the etched surface in accordance with manufacturer instructions. After that, the bracket base was coated with light-cured composite resin (Transbond XT; 3M Unitek, St. Paul, Minnesota, USA) and positioned over the tooth. The bracket was positioned on the mid-buccal surfaces of the teeth, at least 4 mm from the buccal cusp ridges, with the bracket slot perpendicular to the coronal long axis of the tooth, using a bracket positioning gauge. Using a dental probe, the extra composite was removed. All the brackets of both groups were cured using an LED curing light for 10 seconds each from the occlusal, mesial, distal, and gingival aspects. After light curing, specimens were stored in distilled water at 37 °C for 24 hours to allow complete polymerisation of the bonding material.

Evaluation of shear bond strength

**Figure 1:**
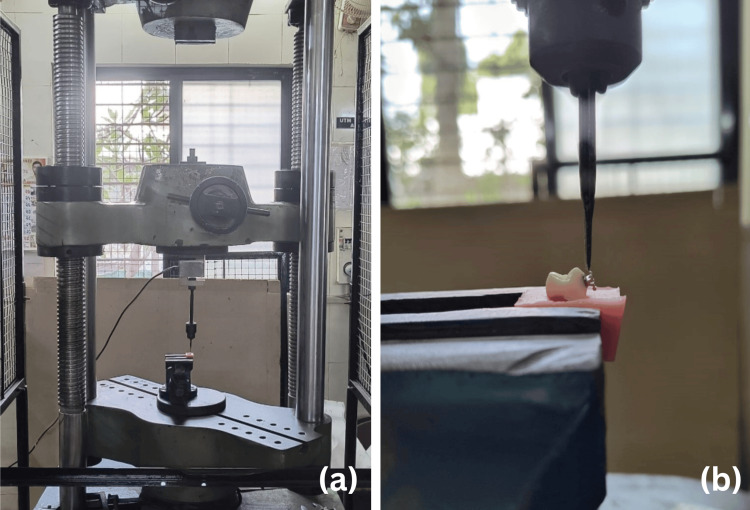
Shear bond strength testing of the sample on a universal testing machine (a) Universal testing machine. (b) Tooth sample loaded on a universal testing machine.

Twenty-four hours after bonding, the specimens were tested for the shear bond strength of the orthodontic brackets. Each specimen was loaded into the lower jig of the universal testing machine for bond strength testing (Figure 1).

The bracket base was positioned in the universal testing machine parallel to the direction of the shear force. A chisel-shaped blade was loaded into the machine's upper section and positioned to make contact with the specimen's bonded area in an occlusogingival direction. A universal testing apparatus with a load cell of 25 KN and a cross-head speed of 1 mm/minute was used to test the shear bond strength. The failure loads (Newtons) were divided by the bracket base's surface area (mm2) to convert the measured shear bond strength values into megapascals (MPa).

Statistical analysis

Statistical analysis was performed using SPSS Statistics version 21 (IBM Corp. Released 2012. IBM SPSS Statistics for Windows, Version 21.0. Armonk, NY: IBM Corp.). Continuous quantitative data was expressed as the mean and standard deviation, respectively. Data normality was checked by using the Shapiro-Wilk test. The significance level was set at p<0.05, indicating that results with a p-value less than 0.05 were considered statistically significant.

## Results

**Figure 2:**
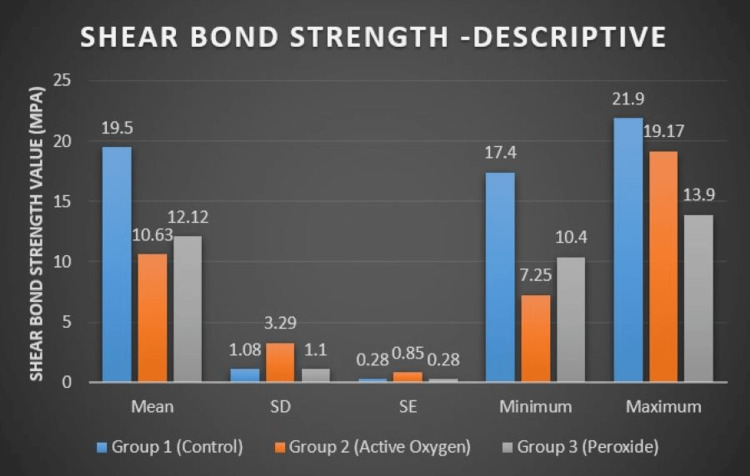
Graphical representation of the mean shear bond strength value (Mpa) among different groups of study SD: standard deviation, SE: standard error, MPa: megapascal

Table 1 and Figure 2 depict the distribution of the effect of different types of bleaching dentifrices (peroxide bleaching dentifrice and active oxygen bleaching dentifrice) on the shear bond strength of orthodontic metal brackets bonded by light-cured composite adhesive to human teeth.

**Table 1:** Mean shear bond strength value (MPa) among different groups of study SD: standard deviation, SE: standard error, MPa: megapascal

	Mean (MPa)	SD	SE	Minimum (MPa)	Maximum (MPa)
Group 1 (control)	19.5	1.08	0.28	17.4	21.9
Group 2 (active oxygen)	10.63	3.29	0.85	7.25	19.17
Group 3 (peroxide)	12.12	1.1	0.28	10.4	13.9

For Group 1, the mean shear bond strength was 19.5 MPa with a standard deviation (SD) of 1.08. The mean shear bond strength of Group 2 was found to be 10.63 MPa with an SD of 3.29. Group 3 had a mean shear bond strength of 12.12 MPa with an SD of 1.1. This shows that Group 1 has the highest shear bond strength, while Group 2 has the least.

Table 2 shows the overall comparative statistics of the effect of different types of bleaching dentifrices (peroxide bleaching dentifrice and active oxygen bleaching dentifrice) on the shear bond strength of orthodontic metal brackets bonded by light-cured composite adhesive to human teeth using the one-way ANOVA "F" test.

**Table 2:** Comparison of the shear bond strength of orthodontic metal brackets within each group p>0.05, no significant difference; *p<0.05, significant; **p<0.001, highly significant SD: standard deviation, MPa: megapascal

	Mean (MPa)	SD	Anova ‘F’ test value	p-value
Group 1 (control)	19.5	1.08	F=76.575	p<0.001**
Group 2 (active oxygen)	10.63	3.29		
Group 3 (peroxide)	12.12	1.1		

On an overall comparison of the shear bond strength of light-cured composite adhesive bonded to teeth treated with different types of bleaching dentifrices, a highly statistically significant (p<0.001) difference was observed among the three groups. Table 3 shows pairwise comparative statistics of the effect of different types of bleaching dentifrices (peroxide bleaching dentifrice and active oxygen bleaching dentifrice) on the shear bond strength of orthodontic metal brackets bonded by light-cured composite adhesive to human teeth using Tukey’s post hoc test.

**Table 3:** Comparison of the shear bond strength of orthodontic metal brackets between three groups p>0.05, statistical significant difference (NS); **p<0.001, high statistical difference, ^p-value calculated using Tukey’s post hoc test

Group	Comparison group	Mean difference	p-value
Group 1 (control) vs.	Group 2 (active oxygen)	8.87	p<0.001**
	Group 3 (peroxide)	7.38	p<0.001**
Group 2 (active oxygen) vs.	Group 3 (peroxide)	1.48	p=0.140 (NS)

Pairwise comparative statistics of shear bond strength (in MPa) among three study groups using Tukey’s post hoc test showed that the difference in shear bond strength of the three groups was of high statistical significance (p<0.001). On comparing the effect of the two dentifrices on the shear bond strength of brackets, the difference was found to be not significant (p=0.140).

## Discussion

The present study aimed to assess the effect on the shear bond strength of metal orthodontic brackets bonded to human teeth treated with different types of bleaching dentifrices. The results showed that treating the tooth with bleaching dentifrices causes a significant reduction in the shear bond strength.

The reason for the reduced bond strength of bleached samples is under debate, but it is suggested that the residual peroxide and oxygen-free radicals within the dental tissues after bleaching dentifrice applications are the primary reason, acting by limiting appropriate polymerisation of the adhesive [6]. The decrease in shear bond strength after hydrogen peroxide bleaching may also be linked to modifications in the surface roughness of the enamel and structural alterations brought on by the loss of prismatic formation, as well as alterations in the organic content, calcium loss, and microhardness [9]. By creating porosities in the outer enamel layer, the bleaching process damages it and causes demineralisation and changes to the calcium-phosphate ratio [10]. Active oxygen bleaching dentifrices work in a similar manner; however, further studies on their mechanism of action and concentration of released oxygen free radicals need to be done.

On comparison among the two dentifrices, the difference was not found to be significant. Brushing with peroxide bleaching dentifrice will lead to a comparatively similar reduction in the bond strength of brackets to the teeth as that of active oxygen bleaching dentifrice. When comparing the two dentifrices to the control group, a significant decrease in shear bond strength was noted.

Various studies have examined the interaction between bleached enamel and the shear bond strength of metal brackets. Shear bond strength has been documented to decrease when brackets are bonded immediately to enamel following bleaching with 35% hydrogen peroxide [8,9,11]. Torres et al., in their study, showed that there is a decrease in the shear bond strength of metal brackets after the use of various whitening dentifrices [2]. Prior to orthodontic treatment, using teeth-whitening toothpaste may cause issues with bracket adherence and, consequently, the therapy that was started. Products that are readily available on the market may be able to whiten teeth and eliminate external stains like silica [12]. Activated charcoal, an abrasive agent, can be included in the formula of a dentifrice to encourage whitening [13]. Additional bleaching agents, like hydrogen peroxide, alter the dentin and enamel's natural colour in a more profound and long-lasting manner [14]. Although the peroxide concentration in these products is lower than the concentration used in trays (home use) or office bleaching techniques, data confirming their oxidant action have already been published, as shown by Hohlen et al. [8].

The results of this study show that the shear bond strength of orthodontic brackets bonded to bleached enamel is significantly reduced. It will be necessary to increase the shear bond strength by using alternative methods such as sandblasting or the application of antioxidants [14-16]. A number of strategies have been put forth to prevent clinical issues resulting from weakened bonds following bleaching. These strategies include removing the enamel's superficial layer, pretreating bleached enamel with alcohol, using adhesives containing organic solvents, and using reducing (antioxidant) agents like glutathione, sodium ascorbate, catalase, and sodium bicarbonate. Some studies suggest delaying bonding after bleaching due to a temporary decrease in bond strength with freshly bleached enamel [17]. A study by Abdel-Hadi et al. found that delaying bracket bonding for five weeks after bleaching significantly increased the bond strength above the accepted range, regardless of the bleaching type [17]. Singh et al. found that adhesive boosters increased the shear bond strength of brackets after bleaching [10].

In both in vitro and in vivo experiments, Mullins et al. found that brackets bonded to recently bleached teeth (10% carbamide peroxide) have an increased likelihood of bond failure. The in vivo investigation found that in the absence of an antioxidant agent, a two-week interval should elapse between bleaching and bonding [18]. According to research by Bulut et al., applying 10% sodium ascorbate to the bleached enamel surface or waiting a week can reverse the decline [19]. A study by Kimyai et al. further elaborated by showing that the application of 10% sodium ascorbate for three hours partially reversed the reduced bond strength following bleaching [20]. It was discovered by Mirhashemi et al. that pretreatment of the surface with Er:YAG and carbon dioxide lasers is more effective than surface pretreatment with Nd:YAG lasers or antioxidant agents for improving the shear bond strength of orthodontic brackets on bleached tooth surfaces [21]. Since this study has shown that there is a significant reduction in bond strength irrespective of the type of bleaching dentifrice used, necessary measures can be taken to minimise the bond failure of brackets.

Limitations and recommendations of the present study

It should be stressed that, as this was an in vitro study, the test circumstances were not exposed to the stresses, temperature swings, changing electrolytes, microbes, or other highly changeable oral cavity elements that characterise the oral environment. Bond strength is affected by a number of substrate-related factors, including tooth type and source, orientation of enamel prisms, age of tooth donors, storage medium and time, bonding area of test samples, operator abilities, and procedure. This means that the findings of this study cannot be applied to the intraoral environment. Nevertheless, despite the inherent limitations of an in vitro study and its inability to fully replicate the complex oral environment, the findings of this research provide valuable insights and serve as a foundational step towards understanding the principles underlying the bond strength dynamics of orthodontic materials.

## Conclusions

The control group had the highest shear bond strength, followed by the peroxide bleaching dentifrice- and active oxygen bleaching dentifrice-treated groups. There was a significant difference in the reduction of shear bond strength of orthodontic brackets bonded to teeth treated with bleaching dentifrice. The observed significant decrease in bond strength highlights a practical concern for orthodontists when planning treatment for patients. Understanding the impact of bleaching dentifrices on bracket bonding is crucial for optimising treatment outcomes and avoiding potential challenges during orthodontic therapy. In light of these findings, it is imperative for orthodontists to routinely inquire about patients' histories of bleaching dentifrice usage during initial consultations. This proactive approach allows clinicians to anticipate and mitigate potential issues related to reduced bond strength, thereby ensuring the stability and efficacy of orthodontic treatment and delivering more predictable and successful orthodontic outcomes.
